# Supplementary material for: Longitudinal Impact of WTC Dust Inhalation on Rat Cardiac Tissue Transcriptomic Profiles
Source: Int J Environ Res Public Health. 2022 Jan 14;19(2):919. doi: 10.3390/ijerph19020919 (PMC8776213; doi:10.3390/ijerph19020919)
Supplement: Supplementary file 1 [file ijerph-19-00919-s001.zip › ijerph-1475352-supplementary.pdf]

Supplementary figures:

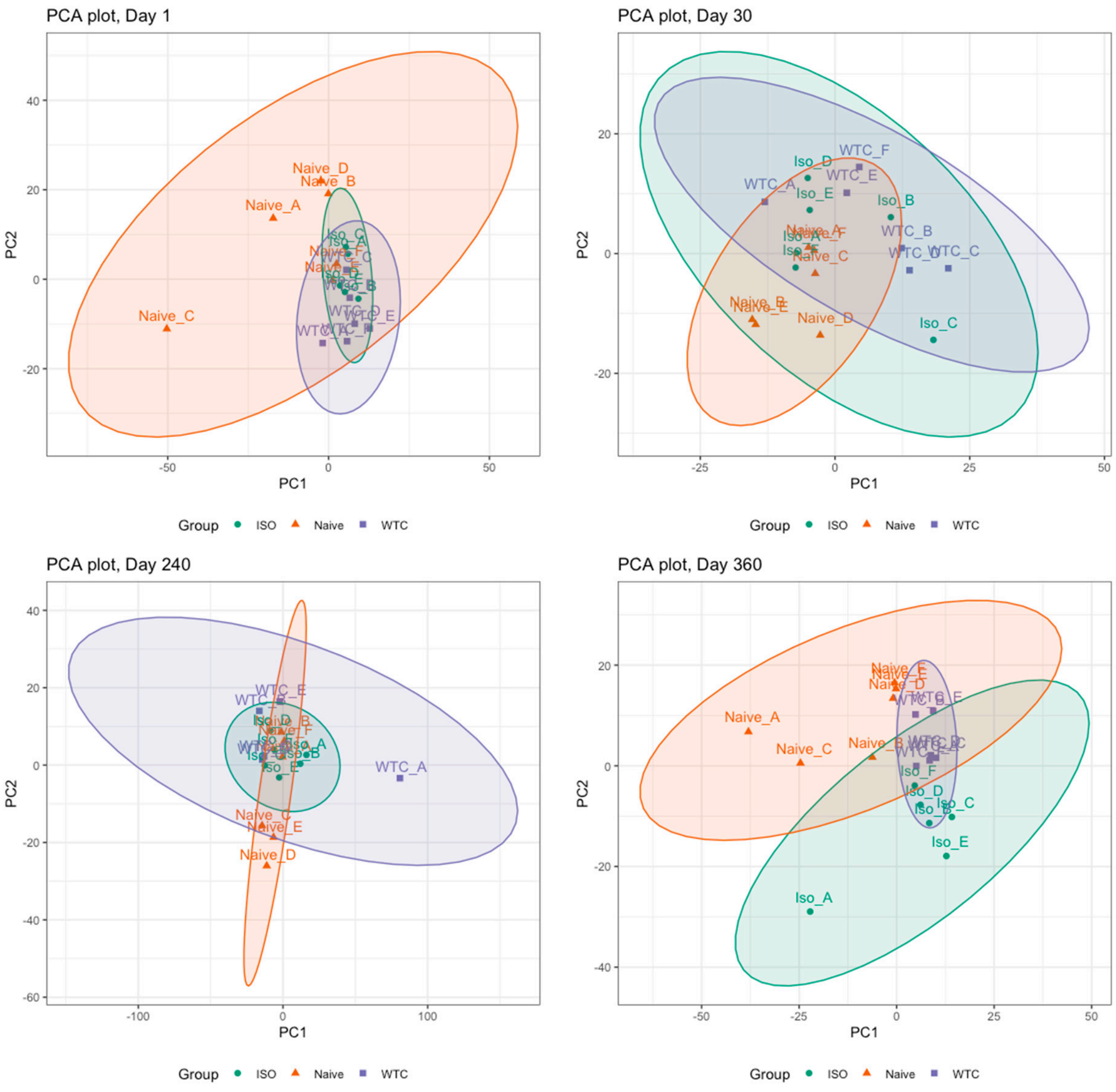

Figure S1. PCA plots to detect possible outlier. PCA was performed on the normalized log-transformed data using all genes (after filtering low expressed genes) for all samples at Days 1, 30, 240 and 360. No evidence of outliers among all the samples at each timepoint was found.

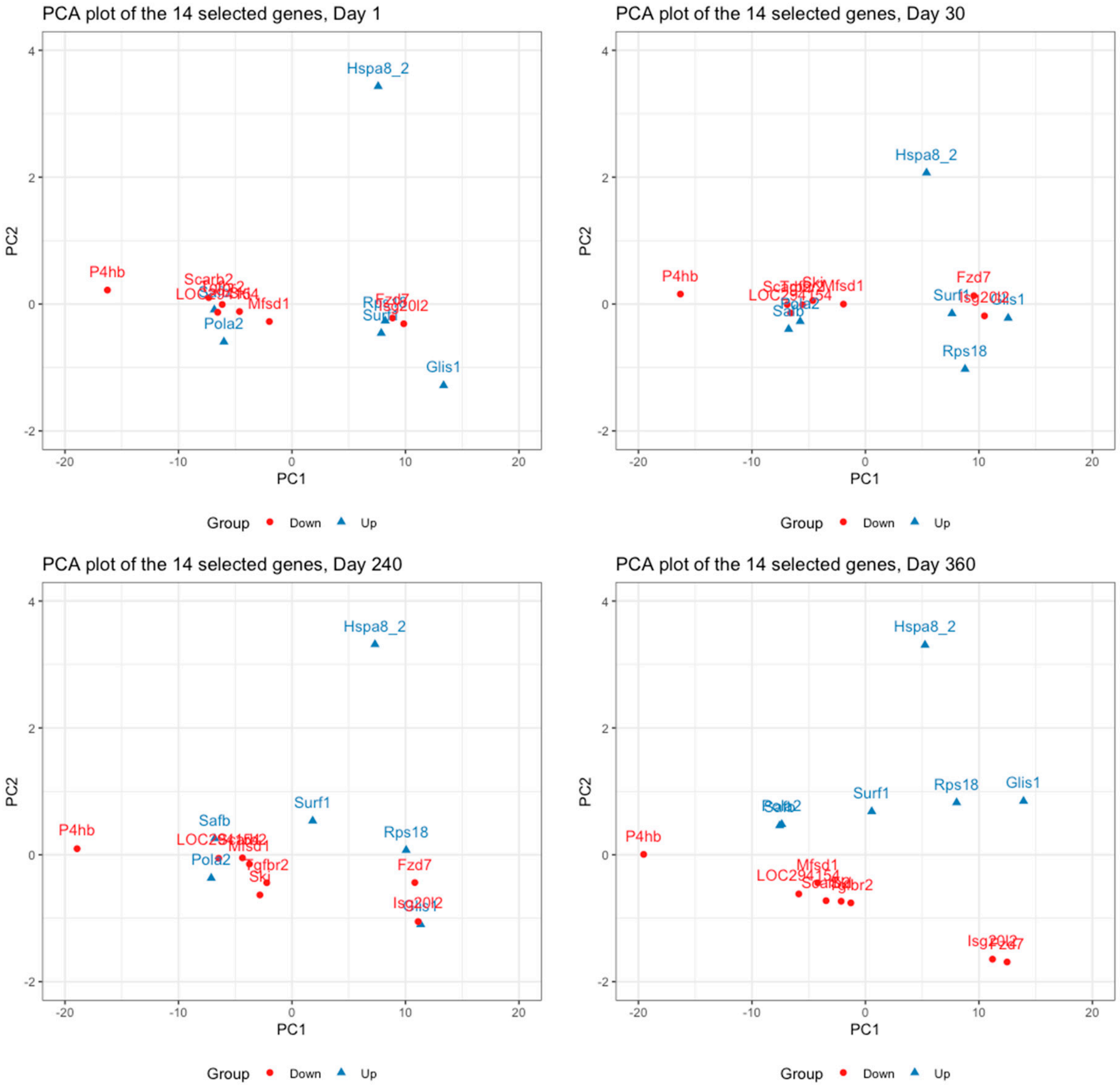

Figure S2. PCA plots of the 14 selected genes at Days 1, 30, 240, and 360. PCA was performed on the normalized log-transformed data of the 14 selected genes (after filtering low expressed genes) using all samples at Days 1, 30, 240 and 360.
